# An exploratory study of the acceptability of indoor residual spraying for malaria control in upper western Ghana

**DOI:** 10.1186/s12889-020-08505-y

**Published:** 2020-04-06

**Authors:** Vitalis Mwinyuri Suuron, Lillian Mwanri, George Tsourtos, Ebenezer Owusu-Addo

**Affiliations:** 1 College of Medicine and Public Health, Flinders University, GPO Box 2100, Adelaide, SA 5001 Australia; 2 Bureau of Integrated Rural Development, Kwame Nkrumah University of Science & Technology, Kumasi, Ghana

**Keywords:** Indoor residual spraying, Malaria, Mosquitoes, Barriers, Householders, Acceptability, Community, Ghana, IRS, Facilitators

## Abstract

**Background:**

Despite the implementation of the World Health Organisation’s recommended indoor residual spraying (IRS) intervention in the upper west region of Ghana to reduce malaria morbidity and mortality, the uptake of this intervention remains low. This study explores the facilitators and barriers to the acceptability and community uptake of indoor residual spraying in a highly endemic region of Ghana.

**Methods:**

The health belief model (HBM) and realist evaluation framework were used to inform the study. A qualitative enquiry was conducted between April to October 2016. Data were collected through focus group discussions and semi-structured interviews with program stakeholders including community members, AngloGold Ashanti malaria control (AGA Mal) spray operators, and AGA Mal officials.

**Results:**

A total of 101 people participated in the study. Considerable barriers to community acceptance of indoor residual spraying (IRS) were found, including, dislike of spray insecticides, inadequate information, religious and cultural beliefs, perceived low efficacy of IRS, difficulties with packing, unprofessional conduct of IRS spray operators, and other operational barriers to spraying. Facilitators of IRS uptake included a perceived effectiveness of IRS in preventing malaria and reducing mosquito bites, incidental benefits, respect for authority, training and capacity building, and sensitization activities.

**Conclusion:**

The numerous barriers to indoor residual spraying acceptance and implications show that acceptance levels could be improved. However, measures are required to address householders’ concerns and streamline operational barriers to increase community uptake of indoor residual spraying.

## Background

Malaria is a disease that is endemic in tropical countries, particularly, in Sub-Saharan Africa. The disease exacts a monumental toll on mankind [1]. In 2015, an estimated 212 million infections were recorded worldwide, with Africa disproportionately accounting for over 90% malaria cases and 394,000 malaria deaths [2]. Ghana is one of the African countries where malaria is endemic, and the entire population is at risk of infection [3]. Despite seasonal variations, malaria is more endemic in the northern part of Ghana, particularly, in the upper western part of the country where a prevalence of 51% was recorded among children of ages 6–59 months [4]. The effects of malaria go beyond health consequences to encompass a reduction in productivity and income loss, which significantly contributes to the slow rate of socio-economic progress in Africa [5].

For this reason, several interventions are being implemented to combat malaria in Sub-Saharan Africa. In Ghana, the interventions being implemented include: targeted indoor residual spraying (IRS), insecticides treated nets (ITNs), intermittent preventive treatment for pregnant women (IPTp), malaria surveillance, and malaria chemotherapy among others. IRS involves the application of insecticides on the inner walls and roofs of houses and domestic animal shelters [6]. The intervention operates by repelling mosquitoes from entering houses and by killing female mosquitoes that rest inside houses after taking their blood meal. In other words, IRS is most effective against endophilic mosquitoes due to their indoor biting and resting habits. Spraying is usually conducted between once and three times a year depending on the type of insecticide and the malaria transmission season [7]. The WHO recommends a number of insecticides for IRS, including: DDT wettable powder (WP), malathion WP, fenitrothion WP, pirimiphos-methyl WP and emulsifiable concentrate (EC), bendiocarb WP and WP in sealed water soluble (WP-SB), propoxur WP, alpha-cypermethrin WP and suspension concentrate (SC) among others [8]. The availability of a wide range of insecticides for IRS facilitates resistance management and enhances the long-term sustainability of malaria vector control [7].

IRS is one of the most effective vector control interventions against malaria [7, 9–12] and recommended by the WHO for adoption either alone or in combination with ITNs in endemic and hyperendemic areas as well as regions prone to malaria epidemic [12]. The intervention was piloted by AngloGold Ashanti malaria control (AGA mal) as a corporate social responsibility programme in the Obuasi Municipality of Ghana in 2006. Susceptibility tests informed the use of the insecticide Pirimiphos-methyl WP for the initial pilot programme [13], although Pro-Guard, Actellic 50 EC and Actellic 300CS were used in subsequent years for the purpose of resistance management [14]. The programme succeeded in reducing malaria by 50% in 2 years [15]. The remarkable success of this programme enabled it secure funding for a scale-up to 40 districts in Ghana; targeting the most endemic regions and districts to enhance the achievement of the then Millennium Development Goals (MDGs) and health equity [16]. The upper west region (UWR) was among the first regions to benefit from the scale-up of IRS spray with the use of Pirimiphos-methyl WP in 2012. However, 2 years after the implementation of this highly effective intervention, malaria persists as a serious public health threat in the upper west region. In 2014, malaria prevalence of 37.8% among children was recorded in the region [17].

Community acceptability is a primary requirement for the successful implementation of vector control interventions, particularly, IRS [18, 19]. Previous survey data demonstrates some level of acceptance of indoor residual spraying in the upper west region of Ghana, with evidence indicating a household coverage of 69.1% [17]. However, this coverage level is below the minimum 80% threshold required for the effectiveness of IRS [20]. The aim of this study is to explore the facilitators and barriers to IRS acceptability in the upper west region of Ghana to help improve community uptake and implementation of IRS in the region. The findings will enable policy makers to design strategies to address identified barriers to IRS uptake and implementation.

## Methods

### Study design and theoretical framework

A qualitative study with an interpretivist approach was adopted to explore phenomena from the perspectives of the householders, AGA Mal officials (implementers of indoor residual spraying), and AGA Mal spray operators. A qualitative interpretivist approach explores the worldview of participants or the meaning they make out of a particular situation [21]. The health belief model provided a framework for exploring householders’ perceptions about the facilitators and barriers to indoor residual spraying acceptability. The health belief model is a value-expectancy theory and holds that people weigh up the cost and benefits of taking recommended preventive health behaviours [22]. The model could, thus, facilitate an understanding of the perceived benefits and barriers to spray acceptance. Given the influence of contextual factors on the outcome of interventions [23, 24], it was deemed necessary to complement the health belief model with a realist evaluation framework to explore the facilitators and barriers to the acceptance and implementation of indoor residual spraying (IRS) intervention. Realist evaluation framework is particularly suited for unpacking how contextual factors and intervention mechanisms or processes influence the outcome [23, 25]. This framework will be useful in providing an understanding of how the underlying mechanisms of IRS implementation and specific contextual factors in the upper west region of Ghana affect the acceptance and implementation of IRS.

### Study setting

Given that malaria is endemic in the entire region, two districts were purposively selected to explore potential rural/urban variations in IRS acceptability. These include Wa Municipal and Daffiama-Busie-Issa (DBI) Districts. The region has a population of 702,110 with Wa Municipals having 107,214 and DBI 32,584 [26, 27]. Climatically, the UWR’ single rainy season starts from April to September with average annual rainfall of about 115 cm. The highest prevalence of malaria is recorded during the rainy season, peaking between June and August. A prolonged dry season follows the rainy season, with the north-east trade winds or harmattan dominating the region with cold and hazy weather from early November to March, and an intense hot weather through to the onset of rainfall in April. The average monthly temperature ranges between 21 °C and 32 °C, with a maximum 40 °C in March [28]. The region’s high temperature facilitates mosquito breeding, resulting in the high prevalence of malaria.

### Data collection

Householders perceptions about the facilitators and barriers to indoor residual spraying (IRS) acceptability were explored. Also, the views of AGA Mal officials and spray operators in relation to the implementation of IRS were explored, and these were particularly relevant because the regimented nature of IRS interventions could influence the outcome of spraying. A purposive sampling technique [29] was used to select heads of households or their representative (aged 18 or above), AGA Mal officials and spray operators to participate in the study [29]. Nine focus group discussions were conducted with householders in four different communities in Wa Municipal and Daffiama-Busie-Issa (DBI) Districts. In addition, two focus group discussions were conducted with AGA Mal spray operators—one each in DBI and Wa Municipal Districts. A sample range between 7 and 10 respondents participated in each focus group as this was deemed pragmatic enough to answer the research questions [30]. Also, eight semi-structured interviews were conducted with AGA Mal officials. Focus groups and interviews are useful methods for exploring individual behaviour [31, 32]. Topic guides relating to key issues for exploration were used to facilitate the focus groups discussions and semi-structured interviews to enhance the collection of comprehensive data [33, 34]. The topic guides were developed based on relevant literature, the theoretical frameworks and the research questions. The topic guide for householders covered their demographic characteristics, and their perceptions about the facilitators and barriers to indoor residual spraying acceptance. Where necessary, questions such as “why wouldn’t some householders spray their houses?” were asked to reduce householders’ hesitation when responding with personal or contentious factors in relation to IRS refusals [18]. On the other hand, the topic guide for AGA Mal officials and spray operators covered their perspectives on the facilitators and barriers to the implementation of indoor residual spraying. Data were collected until the achievement of data saturation, or the point where no new data were being generated [35]. The focus group discussions with householders were conducted in Dagaare, and facilitated by the primary researcher who is competent in the language. The collection of data from multiple sources allowed for data triangulation for a better understanding of findings. This enhances the rigour, validity and credibility of the findings, and thus, increases the trustworthiness of the study [36]. Data were collected between April 2016 to October 2016.

### Ethics

Ethics clearance was received from the Flinders University Social and Behavioural Research Ethics Committee and the Navrongo Health Research Centre institutional review board in accordance with the Nuremberg code of 1948 [37]. Also, informed consent was sought by providing participants with an information pack comprising introductory letters, information sheets and consent forms. In addition, the contents of the information pack were verbally explained to the participants in the local Dagaare language. To ensure participants’ anonymity and confidentiality, no identifying information is provided in the quoted extracts from the interviews and focus groups [38].

### Data management and analysis

Field notes were typed and voice recordings of interviews and focus groups were transcribed verbatim. Focus groups were conducted using the local dialect, and the principal researcher translated the recordings into English. This was facilitated by the fact that the researcher hails from the study setting, and understands the local dialect. The data were analysed using thematic framework analysis. The analytical processes included data familiarisation, coding, identification of thematic framework, indexing, charting and data interpretation [39]. To identify a thematic framework, a descriptive coding was undertaken for each participant group using two householders focus groups transcripts, one AGA Mal spray operators focus groups transcript, and two AGA Mal officials semi-structured interview transcripts. The initial codes were shared with two research Supervisors. The codes underwent a series of refinement during which some codes were merged with input from the Supervisors. A series of iterations were conducted on the codes resulting in the adoption of a final codebook or thematic framework for each participant group. All the transcripts were then uploaded onto NVivo version 11, and a set of codes corresponding with the codebooks or thematic frameworks were created on the software. All the transcripts were then read one by one on Nvivo, and the portions that corresponded with the codes were highlighted or indexed. The highlighted portions were charted onto their corresponding codes on the Nvivo software [40]. The use of the computer based Nvivo software facilitated data storage and retrieval [41]. An integrated approach was adopted in the development of the concepts, code structure, and themes for the purpose of analysis. Consequently, both deductive and inductive approaches to data analysis were adopted [42, 43]. The findings were interpreted from a range of theoretical and conceptual perspectives, especially the Health Belief Model and Realist Evaluation Framework for a broader understanding of the findings. Also, the prior exposure of the primary researcher to the study setting and his participation in IRS implementation provided a positive ‘insider effect’ [44], enhancing the researcher’s understanding of the findings relating to the influence of contextual factors on the acceptance and implementation of IRS. The perspectives of all the authors, and the consensus reached during data analysis added rigour to the interpretation of findings [45].

## Results

### Demographic characteristics of study participants

In total, 101 people—made up of 76 householders, 8 AGA Mal officials, 17 AGA Mal spray operators—participated in the study. Most household participants were aged between 30 and 60 (62.7%), with only a few exceeding 60 years of age (4). Also, almost half (48%) of the household participants had no formal education and 82% of householders were employed in the informal sectors, particularly, farming. All the AGA Mal spray operator were male, and the recruitment of male-only spray operators was said to be a deliberate attempt to avoid the adverse health impact of exposing women to spray insecticides. Finally, the officials of AGA who participated in the study were male-dominated, and aged between 31 and 60 years of age. Table 1 gives a summary of the demographic characteristics of the study participants.

**Table 1:** Demographic characteristics of study participants

Participant groups/variables		Householders	AGA Mal Spray Operators	AGA Mal officials
**Age range**	18–30	21 (28%)	10 (59%)	2 (25%)
	31–60	51 (67%)	7 (41%)	6 (75%)
	61–80	4 (5%)	0 (0%)	0 (0%)
**Sex**	Male	45 (59%)	17 (100%)	7 (87.5%)
	Female	31 (41%)	0 (0%)	1 (12.5%)
**Level of education**	No formal education	36 (48%)	0 (0%)	0 (0%)
	Primary level	10 (13%)	0 (0%)	0 (0%)
	JHS	10 (13%)	3 (18%)	0 (0%)
	SHS	10 (13%)	5 (29%)	0 (0%)
	Tertiary	10 (13%)	9 (53%)	8 (100%)
**Employment status**	Employed (formal)	5 (7%)	17 (100%)	8 (100%)
	Employed (informal)	62 (82%)	0 (0%)	0 (0%)
	Unemployed	8 (10%)	0 (0%)	0 (0%)
	Student	1 (1%)	0 (0%)	0 (0%)

*JHS* Junior High School, *SHS* Senior High School

The themes identified in relation to IRS acceptability by householders were expressed in terms of facilitators and barriers.

### Facilitators of IRS acceptability

#### Perceived effectiveness of indoor residual spraying

Many householders believed that indoor residual spraying (IRS) was effective in preventing malaria, and killing insects such as mosquitoes, flies, mice, cockroaches, spiders and scorpions. This was said to have resulted in a reduction in malaria-related hospital admissions, and hence, enabled householders to continue engaging in important economic activities. Other householders reported that IRS contributed to their general good health. For some other householders, the incidental benefits IRS such as the killing of mice and cockroaches were the primary reason for their acceptance of IRS, and this was corroborated by AGA Mal spray operators. While most householder participants believe that IRS was generally effective in preventing malaria, they perceived recent spray insecticides to be less efficacious in killing mosquitoes and insects. Some householders associated the perceived inefficacy of IRS sprays to the quantity of insecticides that is mixed for spraying, with some blaming spray providers for mixing less insecticides than the standard quantity required, potentially resulting in low dosage. Thus, most householders preferred the previously used insecticides compared to the currently used insecticides. This householder preference was confirmed by AGA Mal officials and spray operators.

“I also want to say something to those responsible for supplying insecticides for spraying that they should send the insecticide they sent some years back. That the previous insecticide is the one that can help us because as for the recent insecticide, it is only called insecticide but it doesn’t do the work of insecticides” [Household participant 10; Daffiama FGD1].

“What they like most is the Pro-guard because of its efficacy, that is the immediate effect because when Pro-guard is used, cockroaches, lizards, rats, even reptiles like snake die instantly, and even houseflies, they die instantly so people prefer that [insecticide] to any other chemical used. The recent one we used, this actellic 300 CS, well, there have been a lot of complains” [AGA Mal official 7].

#### Respect for authority

A few householders acknowledged accepting IRS due to their respect for IRS sprayers as figures of authority or as representatives of government.

“But I see that it’s not only because of the disease; when they say, you should do something, maybe the government gave them the permission, the people doing the spraying have the permission of the government so they were given the right to spray. It is a show of commitment to malaria prevention to spray one’s house but it also demonstrates how obedient householders are to the government and its agencies” [Household participant 1; Sombo FGD1].

#### Education on malaria and malaria prevention

Educational activities by AGA Mal personnel was another important facilitator of IRS acceptability and implementation. Information, education and communications (IEC) personnel routinely educate the public on malaria prevention and IRS while AGA Mal spray operators educate householders during their spraying activities in the communities. Community based volunteers, chiefs, and opinion leaders were all involved in the disseminating information at the community level. For many householders, education on IRS activities through different strategies promoted its acceptance. AGA Mal spray operators regarded education as the main strategy for managing spray refusals, thereby, facilitating the implementation of IRS.

“Mostly in every community we have the advocates who are key people who are known by everyone. So, the stakeholders and advocates are able to spread the information to the others, aside that there are also community forums” [AGA Mal official 5].

“Just the day before the spray day people came to our area; an information van came around to announce about spraying in the evening” [Household participant 7; Konta FGD1].

#### Training and capacity building for personnel

AGA Mal officials and spray operators reported that seasonal training and monthly refresher trainings are conducted for spray operators. According to AGA Mal spray operators and officials, the training is intended to provide education on the importance of spraying to spray operators, as well as equipping them with the technical skills they need to conduct spraying on the walls. Also, the training activities were reportedly intended to develop the communications and data collection skills of spray operators. These were intended to facilitate the collection of spray data, but also to enhance spray operators’ interactions with householders during spraying.

“The management of the district organize them [spray operators], have a training session with them, educate them on the importance of IRS and how they are going to operate when they go to the field … You know, we cannot force them [householders] to accept the programme so they are being trained in such a way that when they go to the community, this and how should talk to the householders in this way or this manner for them to understand the programme so that we can spray their homes for them” [AGA Mal official 3].

### Barriers to indoor residual spraying acceptability

#### Inadequate information dissemination prior to spraying

Inadequate information and education was reported by householders across all the communities as one of the major barriers to the acceptance of spraying, especially, inadequate prior notifications and education about the scheduled spray day. This challenge was attributed to the fact that house to house spray notifications was no longer undertaken by AGA Mal personnel, resulting in householders’ unavailability to accept spraying, or the unexpected arrival of sprayers.

“When they are coming to spray, they don’t send us prior information about the spraying. By the time they come some of us would have gone to the farm or elsewhere so we wouldn’t be around to open our rooms for them to spray. We don’t hear the information that in this month they will be coming to spray or so and that we should try to be around for the spraying. You will just be sitting there and they will come and say, come and move out your belongings for them to spray” [Household participant 5; Daffiama FGD1].

“At first they came to your house and notified you that your house will be sprayed the next day. But now you just sit there and they will appear and say that they have come to spray your house; but you may also just be leaving for work and you cannot go and prepare for spraying. So now the supervisors, the mosquito supervisors are not doing their work” [Household participant 4; Konta FGD1].

#### Inconvenience in packing belongings outside for spraying

The packing, arrangement and rearrangement of householders’ belongings prior to and after spraying was one of the major reported barriers to indoor residual spraying (IRS) acceptability. It was explained that because spraying is conducted on the walls, householders are required to pack some of their belongings outside before sprays can be conducted. However, some householders reported that the workload and time requirement was a significant challenge for them. Also, most AGA Mal spray operators reported difficulties with packing as a challenge to spraying, particularly, in urban communities where householders tend to have a lot of possessions or belongings compared to those in rural communities. It was emphasised that because some communities are larger than others, spray teams sometimes spend two or more days spraying one community. Some householders lamented that sometimes they may pack their belongings outside in readiness for spraying but no spray operator turns up to spray their house on the first day. When this happens, households wishing to spray their houses may need to pack their belongings outside again for the second time on spray-day two, and this affects the acceptance and implementation of indoor residual spraying.

“For some people when they need to prepare their house for spraying, they say they cannot pack their house items to allow the sprayers to spray. Others cannot pack their house items because of the nature of their belongings because the spraying is done on the wall” [Household participant 2; Busie FGD2].

#### Fear and dislike for spray insecticides

Householders were very concerned about the side-effects of spray insecticides. The odour of indoor residual spray (IRS) insecticides was reportedly as one of the contributory factors responsible for refusals. Whereas some householders were concerned about the health implications such as the exposure of asthmatic patients to the odour, other householders associated the odour with inability to sleep. Insecticides odour was also perceived by some householders to be related to the type of insecticides sprayed (some being less favourable than others). Moreover, the debris left on sprayed walls were reported by a few householders as a barrier to spraying. AGA Mal officials and spray operators confirmed householders concerns about the odour of spray insecticides.

“… So yes, you may decorate your room, but the spraying will stain the wall and it’s not appealing to see your room in that way” [Household participant 7; Busie FGD3].

#### Individual religious beliefs and cultural practices

Many householders in rural communities reported that some householders refused IRS spray due to their unwillingness to reveal their traditional religious practices such as the ownership of ‘s*maller gods*’ which were reportedly held in some household rooms. Also, acceptance of IRS in such instances was perceived to be tantamount to exposing the ‘*gods’* to spray insecticides, and this was believed to incur the wrath of the ‘*gods*’, who may inflict punishment on their owners. It was also feared that the spirit of the ‘*gods*’ may flee or die due to spraying, resulting in the loss of the protective powers of the ‘*gods*’. Also, funeral attendance was reported by householders to be one of the main reasons for their inability to accept spraying, especially, when funeral events coincide with the spray day. AGA Mal officials also reported funeral attendance as a barrier to the implementation of IRS. For AGA Mal spray operators, the hanging of charms and amulets on walls and doors by some householders restricted spray activities. In their opinion, because women—who are mostly home when spray men arrive—are not allowed to touch these spiritual objects, spray operators usually conduct spraying while these charms remain hanging on the walls, a situation that was said to be quite worrying to spray operators.

“Because of funerals, I can go to a funeral and by the time I’m back they might have finished spraying my community. Or you may go to the farm early or go to fetch some firewood and by the time you return they have finished spraying” [Household participant 1; Busie FGD1].

“They don’t want the spray men to go in and see the gods, and some of the gods if their shrines are sprayed then there will be serious consequences for the owner. So, they will say that no, you can’t enter that room, that room is never to be entered” [Household participant 1; Daffiama FGD1].

#### Privacy and security

Fears about having the content of ones’ possessions revealed to the public by AGA Mal spray operators was one of the concerns raised by household participants as a barrier to acceptability. This was especially so where the householders perceive their rooms as not being nice enough or where householders perceive their belongings to be in a disorganized manner not befitting of their public image. AGA Mal officials believed that refusals could also stem from householders fears about potential ulterior motives of spray operators.

“Some people do not want the spray men to go into their houses to discover their secrets, so they do not allow the spray men in to spray their houses. Because their house is not nice enough, or because they could not arrange their belongings well; someone can leave their items in a disorderly manner” [Household participant 5; Konta FGD1].

#### Spray operators unprofessional conduct

Householders perceived spray operators’ lack of professionalism as a barrier to indoor residual spraying (IRS) acceptance. There was a perception among householders that the allocation of targets to spray operators resulted in their reluctance to spray once they achieve their targets for the day. The need to achieve targets was said to be the primary motive spurring operators to be selective in the choice of houses to spray, with a preference for bigger houses because such houses are more likely to have many rooms. However, AGA Mal spray operators reported that some householders were indecisive when the spray operators arrived at their house for spraying as they were often asked to come back later in the day when spraying is almost over.

“The last season the spray men came to spray my place … but this year when they came to spray I heard they were around and I went and informed them to come and spray my room but they did not come so that is why my room was not sprayed” [Household participant 3; Daffiama FGD2].

“Sometimes you’ll be there, you will inform them that you have come to spray, but I don’t know, they wouldn’t give you the permission to spray but when you are done and you are about moving to the next house and then they will come and call you and you need to go back to that section to spray, and it makes the work tedious” [AGA Mal spray operator 6; DBI FGD].

#### Operational barriers to spraying

Operational barriers such as limited vehicles and mobility challenges were reported as barriers to the implementation of IRS. AGA Mal officials reported that the use of limited vehicles affected the transportation of spray operators from operational sites to the communities for spraying. According to the officials, the rainy season compounded the difficulties involved in transporting spray operators to rural communities due to the bad nature of the road network. AGA Mal spray operators reported congestion in buses and the transportation of spray operators in batches as some of the challenges they encounter. These challenges reportedly led to a late arrival of spray operators in rural communities when most householders may have gone to their farms, especially, during the rainy season. Spray operators were also concerned about the difficulties involved in carrying their spray logistics such as spray pump, insecticides, and personal protective equipment from house to house to conduct spraying in the communities, especially, in communities with dispersed housing. Moreover, frequent breakdown of spray equipment was reported by both AGA Mal officials and spray operators as a barrier to spraying.

“Most of the times we are always many in the bus, sometimes the bus has to go and come two times or even more than three times sometimes. When we are going to a far village, by the time the second bus gets there the first bus operators have already finished, and even by then you get there and the people have already gone to farm … There is also another challenge with carrying of our equipment, it’s like you have to carry your bag, your bucket, your pump. This is a lot; it’s always a lot for us to carry these from one house to another” [AGA Mal spray operator 7; DBI FGD].

“One other challenge we have is the frequent breakdown of our spray equipment, the spray pump. These are things that are not manufactured here in Ghana; they are imported without the necessary spare parts for replacement. It affects the spraying in a sense that, when the equipment is not working you lose production for that day” [AGA Mal official 2].

## Discussions

This study was conducted to explore the barriers and facilitators to indoor residual spraying (IRS) acceptability in the Upper West region of Ghana. The study found that most households accepted IRS spraying due to their perceived effectiveness in killing mosquitoes and insects, and reducing malaria infections. Similarly, AGA Mal officials and spray operators reported that benefits of spraying, particularly, the perceived effectiveness of sprays in killing domestic insects motivated householders to accept spraying. Similar findings have been reported in Ethiopia [46] and South Africa [47] where respondents perceived IRS to be effective in killing mosquitoes and insects, as well as preventing malaria. These findings are in consonance with the health belief model which associates the acceptability of an intervention with the perceived benefits of that intervention [48]. However, householders’ acceptance of spraying primarily due to the unintended benefits spraying other than for malaria prevention, poses a long-term challenge to the sustainability of spray acceptance in the medium to long-term. This is because spray insecticides may be effective in preventing malaria but overtime become ineffective in killing insects and domestic pests due to resistance development. In such instances, spray acceptance may become a challenge for householders who mainly accept spraying due to the efficacy of spraying in killing insects and domestic pests [49].

In most householders’ opinion, education activities relating to malaria prevention, and especially, the provision of pre-spray notifications or sensitizations helped create awareness about the scheduled spray day, ensuring that householders avail themselves for spraying on the spray day. In the same vein, AGA Mal officials and spray operators regarded education activities as one of the key facilitators of IRS implementation. These findings are in conformity with the realist evaluation framework [23] because the mechanisms of IRS such as pre-spray education and sensitization are essential to the outcome of spraying, and thus, facilitates the implementation of IRS intervention. Education activities are also key to triggering householders’ cues to action [23], enhancing their acceptance of IRS. The importance of education activities to spray acceptance and implementation highlights the potential negative impact of inadequate education on spray outcome. This is because inadequate information or pre-spray notifications could affect awareness about spraying, resulting in low acceptability.

A few householders were motivated to accept spraying due to their respect for authority or government which is consistent with previous studies. Montgomery, Munguambe and Pool [50] found that a perceived responsibility to fulfil one’s national obligations was one of the main facilitators of IRS acceptance in Mozambique. The perceived national obligation reportedly enhanced spraying by inspiring a respect for government health agencies, or fear of punishment resulting from spray refusal. However, the acceptance of spraying based on government authority may be contrary to the ethics of public health which advocates for individual autonomy in decision making relating to the acceptance of interventions [50].

Also, the findings of the study indicate a seasonal variation in IRS acceptance in rural communities due to households’ early departure to their farms, and the challenges with transporting spray providers to rural communities. Because most hard-to-reach communities have bad road network, transporting spray operators to these communities may be very challenging during the rainy season, increasing the travel time. This finding could be interpreted in the light of realist evaluation framework which posits that contextual factors trigger different mechanisms to produce programme outcomes [23]. In the current study, contextual factors such as bad road network and inadequate transport affected sprayers motivation, and resulted in their late arrivals in rural and hard-to-reach communities which impeded the acceptance and implementation of house spraying intervention. Thaddeus and Maine's [51] three-phase delay framework conceptualises that physical accessibility difficulties such as availability of transportation and travel time have a negative impact on access to health care. Hence, the delay of spray operators may lead to the non-coverage of some hard-to-reach communities with IRS spray. Ellis [52] argues that resource constraints and the need to demonstrate short term results may lead to the concentration of efforts in easy to reach populations at the expense of hard-to-reach communities. In his view, when dealing with hard-to-reach populations, providers often risk over-providing to those who are easiest to reach while at the same time under-providing for those difficult to reach. To avert this, measures are required to enhance the implementation of IRS in rural and hard-to-reach communities in conformity with the principles of equity and social justice as espoused by the Alma Ata declaration on primary health care [53]. IRS spray schedules may need to be designed in such a way that hard-to-reach communities are sprayed in the initial stages of spraying (April/May) before the rain intensifies. Although the unavailability of households for spraying is not unknown in the literature [54, 55], the current findings indicate how transportation challenges indirectly contributes to householders unavailability to accept spraying in rural communities.

The findings indicate that most households were concerned about the side-effects of spraying, including skin irritation, breathing difficulties, inability to sleep, odour and staining of walls. Similar findings were documented by studies in Tanzania where spray insecticides were reportedly perceived to be responsible for causing skin rashes, stomach upsets, swollen face, damage to internal organs, and death following prolonged exposure [56]. In Mexico, Rodriguez and colleagues [19] reported that spray insecticides were perceived to cause irritation and dizziness. Interestingly, this current study’s finding of a perceived inability to sleep or eat due to spray insecticides are important new additions. The odour of spray insecticides may have featured prominently in the current study because most houses in the rural communities of the Upper West region have smaller windows or no windows for ventilation. Realist framework holds the assumption that the context and mechanisms of interventions have an influence on the outcome of the interventions [57]. This presupposes that spray outcomes from households’ whose rooms have less ventilation may vary from those with more ventilation. Therefore, malaria control programmes may need to adopt suitable systems and practices for special contexts such as housing structures that do not have sources of ventilation. This will help reduce the bad smell of insecticides in less ventilated rooms following spraying, and help increase community acceptance of spraying. More importantly, efforts should be made towards introducing an odour-less insecticide for IRS spraying as this may be better tolerated by householders. Further, there is the need for AGA Mal to strengthen its existing partnership with the Ghana health service to promptly respond to any side-effects of spraying.

Moreover, individual religious and cultural beliefs such as the ownership of ‘*smaller gods*’ was found to constitute a barrier to spraying in rural communities. These findings are not dissimilar to previous studies. Munguambe et al. [58] observed that spirit houses in traditional healers’ homes were prevented from being sprayed in Mozambique. Elsewhere, spray insecticides were perceived to anger or kill the spirits of the ‘*gods’* [59]. The health belief model posits that the higher the perceived susceptibility to infection the more likely it is for individuals to accept preventive interventions [60]. Given the role of religious beliefs in influencing disease interpretation, an individual’s dependence on ‘*smaller gods*’ for spiritual protection could result in the perception that one is less susceptible to malaria infection, thereby, affecting the acceptance of indoor residual spraying. Furthermore, the reciprocal nature of funerals [61] imply that funeral attendance may not only expose householders to malaria infection, but also inhibit their cues to action [62], since most householders may have a preference for attending funerals rather than having their homes sprayed on schedule.

Evidence from the study showed household concern about privacy and security due to the entry by spray operators into people’s houses to conduct spraying. Some household participants expressed their worry about the possibility of having the contents of their belongings revealed to the public by some unscrupulous spray operators. A similar study in Tanzania reported that the fear of domestic intrusion led to the perception that spray operators were spying on people’s lives [55], while a similar concern in Rwanda resulted in some householders refusal to spray their bedrooms and storage areas [63]. In a sociological analysis of the impact of vector control at the household level, some scholars argued that the entry into homes by uniformed male spray operators not only extends the power of public institutions into the domestic front, but also raises serious security concerns since the householders present during the day—when spraying is conducted— are mostly women and children [64]. Coimbra [65] contends that the use of paramilitary terms such as combat and campaign by malaria vector control personnel does not help because householders may be unwilling to allow a ‘battle’ in their homes, even if the ‘war’ is being fought for their benefit. Although householders’ privacy and security concerns may be unavoidable, these concerns may be minimized by increasing the involvement of highly respected community members and opinion leaders in IRS implementation.

Another important finding in this study is that the house preparations required for spraying was identified as a major barrier to IRS acceptance and implementation, particularly in urban communities. This could be understood because the perceived barrier to spray acceptance [47, 62] such as packing household belongings outside for spraying may discourage some householders from spraying their rooms. Also, the context of interventions [66] such as urban areas where householders tend to own more possessions could affect the implementation of IRS. Although similar findings have been reported earlier [47, 56], the current study introduces a rural/urban dynamics in terms of how packing affects the acceptance and implementation of IRS.

## Conclusions

Because non-spraying did not always stem from deliberate refusals, this implies that the level of indoor residual spraying (IRS) acceptance could be improved if adequate measures are put in place to address householders’ concerns and streamline operational barriers. AGA Mal may need to intensify community education to correct the wrong perception that IRS is not effective in killing mosquitoes and other insects. Moreover, the adoption of an integrated approach to malaria control that seeks to address the broader social determinants of health such as empowerment of communities could help enhance the acceptability of spraying. In addition, addressing other logistical barriers to implementation such as inadequate vehicles need to be addressed to facilitate the implementation of IRS. The introduction of odourless spray insecticides that have a longer period of efficacy could allay household concerns about the side-effects of spraying, and reduce the burden of preparing one’s home for spraying.

## Data Availability

The dataset used during the current study are not publicly available due to the data management restrictions imposed by the social and behavioural research ethics committee of Flinders University, but available from the corresponding author on reasonable request.

## Abbreviations

IRS: Indoor residual spraying
HBM: Health belief model
AGA Mal: AngloGold Ashanti malaria control
ITNs: Long lasting insecticide treated nets
MDGs: Millennium development goals
DBI: Daffiama-Busie-Issa District
UWR: Upper west region
JHS: Junior high school
SHS: Senior high school
IEC: Information, education & communication
FGD: Focus group discussion
WHO: World health organization
WP: Wettable powder
EC: Emulsifiable concentrate
CS: Suspension concentrate
WP-SB: Wettable powder in sealed water soluble
